# Patient-Reported Adverse Events and Early Treatment Discontinuation Among Patients With Multiple Myeloma

**DOI:** 10.1001/jamanetworkopen.2024.3854

**Published:** 2024-03-27

**Authors:** John Devin Peipert, Fengmin Zhao, Ju-Whei Lee, Shu-en Shen, Edward Ip, Nathaniel O’Connell, Ruth C. Carlos, Noah Graham, Mary Lou Smith, Ilana F. Gareen, Pamela J. Raper, Matthias Weiss, Shaji K. Kumar, S. Vincent Rajkumar, David Cella, Robert Gray, Lynne I. Wagner

**Affiliations:** 1 Department of Medical Social Sciences, Northwestern University Feinberg School of Medicine, Chicago, Illinois; 2 Dana Farber Cancer Institute, ECOG-ACRIN Biostatistics Center, Boston, Massachusetts; 3 Department of Biostatistics and Data Science, Wake Forest University School of Medicine, Winston-Salem, North Carolina; 4 Univeristy of Michigan Comprehensive Cancer Center, Ann Arbor; 5 Research Advocacy Network, Chicago, Illinois; 6 Department of Epidemiology and the Center for Statistical Sciences, Brown University School of Public Health, Providence, Rhode Island; 7 Gillings School of Global Public Health, Department of Health Policy and Management, University of North Carolina at Chapel Hill; 8 ThedaCare Cancer Center, Appleton, Wisconsin; 9 Mayo Clinic, Rochester, Minnesota

## Abstract

**Importance:**

There is substantial interest in capturing cancer treatment tolerability from the patient’s perspective using patient-reported outcomes (PROs).

**Objective:**

To examine whether a PRO question, item 5 from the Functional Assessment of Cancer Therapy-General General Physical Wellbeing Scale (GP5), was associated with early treatment discontinuation (ETD) due to adverse events.

**Design, Setting, and Participants:**

This prospective survey study was conducted from February to April 2023. Among participants in the ECOG-ACRIN E1A11 trial (a phase 3, parallel design trial conducted between 2013 and 2019), patients with newly diagnosed multiple myeloma were randomized to receive bortezomib (VRd) or carfilzomib (KRd) plus lenalidomide and dexamethasone as induction therapy. The GP5 item was administered at baseline (pretreatment) and at 1 month, 2.8 months, and 5.5 months postbaseline. Eligible participants included patients with newly diagnosed multiple myeloma treated at community oncology practices or academic medical centers in the US.

**Exposures:**

GP5 response options were “very much,” “quite a bit,” “somewhat,” “a little bit,” and “not at all.” Responses at each assessment while undergoing treatment (1 month, 2.8 months, and 5.5 months) were categorized as high adverse event bother (ie, “very much,” and “quite a bit”) and low adverse event bother (ie, “somewhat,” “a little bit,” or “not at all”). In addition, change from baseline to each assessment while undergoing treatment was calculated and categorized as worsening by 1 response category and 2 or more response categories.

**Main Outcome and Measure:**

ETD due to adverse events (yes vs no) was analyzed using logistic regression adjusting for treatment group, performance status, gender, race, and disease stage.

**Results:**

Of the 1087 participants in the original trial, 1058 (mean [SD] age 64 [9] years; 531 receiving VrD [50.2%]; 527 receiving KRd [49.8%]) responded to item GP5 and were included in the secondary analysis. A small proportion (142 patients [13.4%]) discontinued treatment early due to AEs. For those with high adverse-effect bother, GP5 while undergoing treatment was associated with ETD at 1 month (adjusted odds ratio [aOR], 2.20; 95% CI, 1.25-3.89), 2.8 months (aOR, 3.41; 95% CI, 2.01-5.80), and 5.5 months (aOR, 4.66; 95% CI, 1.69-12.83). Worsening by 2 or more response categories on the GP5 was associated with ETD at 2.8 months (aOR, 3.02; 95% CI, 1.64-5.54) and 5.5 months (aOR, 5.49; 95% CI, 1.45-20.76).

**Conclusions and Relevance:**

In this survey study of the E1A11 trial, worse GP5 response was associated with ETD. These findings suggest that simple assessment of adverse-effect bother while receiving treatment is an efficient way to indicate treatment tolerability and ETD risk.

## Key Points

**Question:**

Are direct reports from patients about their treatment’s adverse effects associated with early treatment discontinuation?

**Findings:**

In this survey study with 1058 participants in the ECOG-ACRIN E1A11 trial, with newly diagnosed multiple myeloma, when captured during treatment, a single question from the Functional Assessment of Cancer Therapy (“I am bothered by side effects of treatment”) was significantly associated with subsequent discontinuation of treatment due to adverse events.

**Meaning:**

These findings suggest that brief, single-question assessments accurately reflect patients’ ability to tolerate treatment and may be an efficient way to assess risk for treatment discontinuation due to adverse events or compare tolerability of different treatments.

## Teaser

This secondary analysis of the ECOG-ACRIN E1A11 trial analyzes the association of patient-reported adverse effects with early treatment discontinuation in patients with multiple myeloma.

## Introduction

Patient-reported outcome (PRO) measures have been included as end points in cancer clinical trials for decades. PROs can capture multiple concepts relevant to evaluation of cancer treatments, including health-related quality of life (HRQoL), disease symptoms, and adverse effects of treatment. Recently, interest has increased in capturing treatment tolerability from the patient’s perspective.^1,2,3^ This interest reflects a shift away from using clinician-assessed adverse events (AEs; eg, Common Terminology Criteria for Adverse Events [CTCAE]^4^) as the sole approach to determining if a treatment is tolerable. A recent Friends of Cancer Research white paper recommended that “[a] complete understanding of tolerability should include direct measurement from the patient on how they are feeling and functioning while on treatment.”^1^ Aligned with this recommendation, in their recently published, *Core Patient-Reported Outcomes in Cancer Clinical Trials: Guidance for Industry*,^5^ the US Food and Drug Administration (FDA) identified at least 2 PRO concepts directly associated with treatment tolerability: overall AE impact and symptomatic adverse events (AEs).

The Functional Assessment of Cancer Therapy (FACT) measurement system is among the most commonly used sources of PROs in cancer trials,^6,7^ especially the overall HRQoL core score (ie, the FACT–General [FACT-G]).^8^ The FACT-G is widely used because it is included in many tumor-specific FACT instruments (eg, FACT-Breast and FACT-Colon). The recent interest in patient-reported treatment tolerability has drawn substantial attention to 1 of the 7 items included in the FACT-G Physical Well-Being scale (item GP5). GP5, “I am bothered by side effects of treatment,” is rated on a 5-point scale ranging from “not at all” (0) to “very much” (4). The GP5 item has been put forth as a promising approach for capturing overall AE impact.^5^ Because item GP5 asks generally about “side effects,” it has the potential to capture the overall impact of a broad range of cancer-treatment AEs, allowing potential comparisons within and between cancer and treatment types and is able to capture the global impact of AEs, an area of measurement in treatment tolerability identified by an FDA and Critical Path Institute workshop^2^ as highly important.

The use of GP5 has been supported by previous studies^9,10^ examining its measurement properties, with validity studies finding associations with number of AEs and overall HRQoL across a range of cancer types and in both clinical trial and nontrial settings in the US and internationally. In addition, GP5 measured at baseline has been associated with subsequent early treatment discontinuation.^11^ Additional research is needed to understand the ability of GP5 to capture the effects of increasing toxicity within the setting of a clinical trial while patients are receiving treatment. To investigate this question, we analyzed data from the ENDURANCE trial from the ECOG-ACRIN Cancer Research Group (E1A11)^12^ to determine whether worsening AE bother measured by GP5 during the trial was associated with treatment discontinuation due to AEs.

## Methods

### The E1A11 Trial

This survey study was deemed exempt from institutional review board approval from Northwestern University because it was a secondary analysis of deidentified data in accordance with the Common Rule. We selected the E1A11 trial (ENDURANCE)^12^ for a post hoc analysis based on the following criteria: (1) phase 3 therapeutic trials conducted by the ECOG-ACRIN Cancer Research Group; (2) trial treatment regimen included at least 1 group containing a targeted therapy; and (3) the FACT-G item GP5 was administered prior to initiation of protocol therapy and at multiple time points during treatment. In E1A11,^12^ ethical approval was obtained by the National Cancer Institute central institutional review board, local independent ethics committees, and institutional review boards at each participating site. All patients provided written informed consent.

E1A11^12^ is a phase 3, parallel design trial that randomized patients with newly diagnosed, standard-risk multiple myeloma to receive either a bortezomib, lenalidomide, and dexamethasone (VRd) regimen or carfilzomib, lenalidomide, and dexamethasone (KRd) regimen as induction therapy, and then administered either limited or indefinite lenalidomide as maintenance. In the induction phase, treatment was received for twelve 3-week cycles (VRd group) or nine 4-week cycles (KRd group). Only data from the induction phase were used for this study. The primary end point for the induction phase was progression-free survival. Scales from the FACT Gynecologic Oncology Group–Neurotoxicity (FACT-NTx) and FACT-Multiple Myeloma (FACT-MM) instruments were among the secondary end points. Both the FACT-NTx and FACT-MM include the FACT-G Physical Well-Being scale, which in turn includes the GP5 item. They were administered at baseline (pretreatment) and at 1 month, 2.8 months, 5.5 months, and 8.3 months postbaseline while patients were receiving treatment. The overlapping content of the FACT-NTx and FACT-MM were only administered once. We focused only on the 1-, 2.8-, and 5.5-month assessments because the 8.3-month assessment was at the end of treatment. Eligible patients were adults (≥18 years) with newly diagnosed, standard or intermediate risk multiple myeloma who were not eligible for autologous stem cell transplantation and with an Eastern Cooperative Oncology Group performance status rating (ECOG PSR) of 0 to 2. Previous treatment was permitted up to 1 cycle (≤4 weeks) of previous chemotherapy with no more than 160 mg of dexamethasone and no previous lenalidomide, bortezomib, or carfilzomib. Previous radiotherapy was permitted if there was no residual toxic effect related to radiation. The full eligibility criteria are published elsewhere.^12^

A total of 1087 patients were randomized to treatment in the ENDURANCE trial (542 to VRd and 545 to KRd).^12^ Of these, 1058 had any FACT-NTx or FACT-MM data at any assessment time point (1040 at baseline, 961 at 1 month, 895 at 2.8 months, and 635 at 5.5 months).^12^ The median (IQR) duration of induction treatment for treated patients was 7.1 (3.4-8.9) months in the VRd group and 8.5 (5.0-9.1) months for the KRd group.^12^ Among the enrolled population, 110 (10.1%) had prior radiation therapy, 251 (23.1%) had prior treatment with corticosteroids, 149 (13.7%) had prior treatment with bisphosphonates, and 17 (1.6%) had another prior treatment.^12^

### Measures

The primary independent variable of interest for this study was the FACT-G item GP5. We derived several indicators of overall AE impact while undergoing treatment (postbaseline) and change in overall AE impact using the GP5. First, based on work from a previous study,^11^ we dichotomized GP5 while undergoing treatment as high AE bother (ie, responses of “very much” [4] and “quite a bit” [3]) and low bother (ie, responses of “somewhat” [2], “a little bit” [1], or “not at all” [0]). Next, we created an indicator for the maximum GP5 while undergoing treatment, defined as the highest (worst) GP5 value at any GP5 assessment while undergoing treatment. We also applied a baseline adjustment to the maximum GP5 while undergoing treatment using previously published methods wherein the maximum GP5 while undergoing treatment response was used if the maximum response was higher (worse bother) than the patient’s baseline GP5, or a value of 0 (representing “not at all”) was given if the maximum GP5 response was the same or better than baseline; this approach was first used with the PRO version of the CTCAE (PRO-CTCAE).^13,14^ We calculated the change in GP5 from baseline to each GP5 assessment while undergoing treatment, as well as change from baseline to the maximum GP5 response while undergoing treatment, as the difference between the response while undergoing treatment and the baseline response. Each of the GP5 change indicators was then categorized as (1) stayed the same or improved, (2) worsened by 1 response category, or (3) worsened by 2 or more response categories.

When a patient discontinued induction treatment early, the reason was captured on the posttreatment clinical report form per study protocol. Predefined reasons for early discontinuation included alternative therapy, AE, disease progression, patient withdrawal or refusal, death, other complicating disease, physician decision, and noncompliance. The primary outcome of the present study was early discontinuation of treatment due to AE (yes vs no) in E1A11. Each patient was coded as having discontinued early for AE or not (including early discontinuation due to other reasons and completion of induction therapy per protocol). In addition, we used several patient characteristics, including baseline ECOG PSR, gender, race, and International Staging System (ISS) stage to explain discontinuation. Race categories included American Indian or Alaska Native, Asian, Black or African American, Native Hawaiian or Other Pacific Islander, White, unknown, and not reported. Race was included to fully account for the diversity of participating patients.

### Statistical Analyses

We included randomized patients with complete GP5 responses at any assessment time point. For all statistical tests, a nominal 2-sided *P* < .05 or 95% CI not crossing 1 was considered statistically significant. Because this study was exploratory, no adjustment was made for multiple comparisons. Missingness was defined as having completed other FACT-NTx or FACT-MM items but having skipped the GP5. Missing data was handled with complete case analysis. All analyses were conducted in SAS version 9.4 (SAS Institute).

We summarized patient characteristics with frequencies and percentages or means, SDs, and ranges, as appropriate. We visualized distributions of GP5 responses at each time point, including whether GP5 was missing, using a stacked bar chart. We visualized change in GP5 response across assessment time points using a Sankey bar chart with the SAS Sankey macro.^15^ The x-axis of the Sankey bar chart has a stacked bar chart for each assessment time point. The y-axis shows the proportion of patients at each time point with each GP5 response option. In between each bar chart is a band varying in thickness to show how many patients moved from each GP5 response option to each other between each assessment time point.

We examined associations of the GP5 while undergoing treatment and GP5 change indicators with early discontinuation of treatment due to AEs using logistic regression analyses. For any given model, only patients who completed treatment up to the follow-up time point were included in the analysis. For all analyses, we omitted patients who reported “very much” on GP5 at baseline (13 participants) because they were already at or near the ceiling. We fit separate models for each GP5 while undergoing treatment and GP5 change indicator. First, we estimated unadjusted odds ratios (ORs) for this association. Then we added covariates to the models, including treatment group (reference, KRd), age (entered as continuous), baseline ECOG PSR (reference, 0), gender (reference, woman), race (reference, White), and ISS stage (reference, Stage I). As a sensitivity analysis to account for the effect of baseline GP5, we fit a set of logistic regression models adding in the baseline GP5 value as a covariate (entered as a trend); these analyses did not exclude patients who reported “very much” at baseline. We fit a model for each GP5 while undergoing treatment and GP5 change indicator separately as the outcome, and these models only adjusted for baseline GP5. We then added to each of these models the set of covariates described above. Data analysis was conducted from February to April 2023.

## Results

Of the 1087 patients in the original trial,^12^ 1058 (mean [SD] age 64 [9] years; 626 male [59.2%]; 7 American Indian or Alaska Native [0.7%]; 16 Asian [1.5%]; 124 Black or African American [11.7%]; 1 Native Hawaiian or Other Pacific Islander [0.1%]; 867 White [82.0%]; 24 race not reported [2.2%]; 19 race unknown [1.8%]) had the GP5 completed (988 of 1040 at baseline [95.0%]; 953 of 961 at 1 month [99.2%]; 895 of 895 at 2.8 months [100.0%]; and 631 of 635 at 5.5 months [99.4%]). Of the 988 patients with nonmissing GP5 at baseline, 13 (1.3%) gave a response of “very much,” 22 (2.2%) gave a response of “quite a bit,” 58 (5.9%) gave a response of “somewhat,” and 86 (8.7%) gave a response of “a little bit”. Characteristics of those with GP5 responses at any time point are given in Table 1. The proportions of patients in each treatment group were equal in this analysis sample (531 receiving VRd [50.2%] and 527 receiving KRd [49.8%]). The largest proportions had an ECOG PSR status of 1 (510 participants [48.2%]). Equal proportions of participants were in ISS stage I (392 participants [37.3%]) and stage II (384 participants [36.5%]). A minority (142 participants [13.4%]) discontinued treatment early due to AEs.

**Table 1.** Patient Characteristics

Characteristic	Patients, No. (%) (N = 1058)
Treatment group	
VRd	531 (50.2)
KRd	527 (49.8)
Baseline Eastern Cooperative Oncology Group performance status	
0	441 (41.7)
1	510 (48.2)
2	89 (8.4)
3	18 (1.7)
Age, mean (SD) [range], y	64 (9) [32-88]
Gender	
Woman	432 (40.8)
Man	626 (59.2)
Race	
American Indian or Alaska Native	7 (0.7)
Asian	16 (1.5)
Black or African American	124 (11.7)
Native Hawaiian or Other Pacific Islander	1 (0.1)
White	867 (82.0)
Not reported	24 (2.2)
Unknown	19 (1.8)
International Staging System Stage	
I	392 (37.3)
II	384 (36.5)
III	275 (26.2)
Reason for early discontinuation	
Alternative therapy	168 (15.9)
Adverse event	142 (13.4)
Disease progression	48 (4.5)
Patient withdrawal or refusal	59 (5.6)
Death	21 (2.0)
Other complicating disease	16 (1.5)
Physician decision	12 (1.1)
Noncompliance	10 (0.9)
Other	34 (3.2)

Abbreviations: KRd, carfilzomib, lenalidomide, and dexamethasone; VRd, bortezomib, lenalidomide, and dexamethasone.

**Figure 1.**
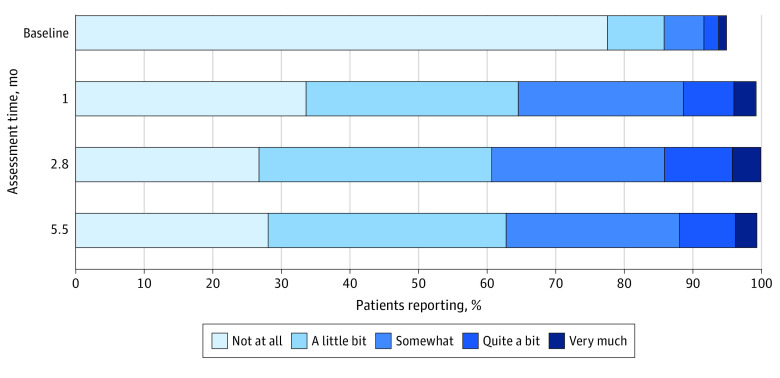
Frequency of GP5 Responses by Assessment Time Point The GP5 refers to item 5 of the Functional Assessment of Cancer Therapy-General. The sample sizes at each assessment time point included 988 patients at baseline, 953 patients at month, 895 patients at 2.8 months, and 631 patients at 5.5 months. Bars do not reach 100% due to missingness.

**Figure 2.**
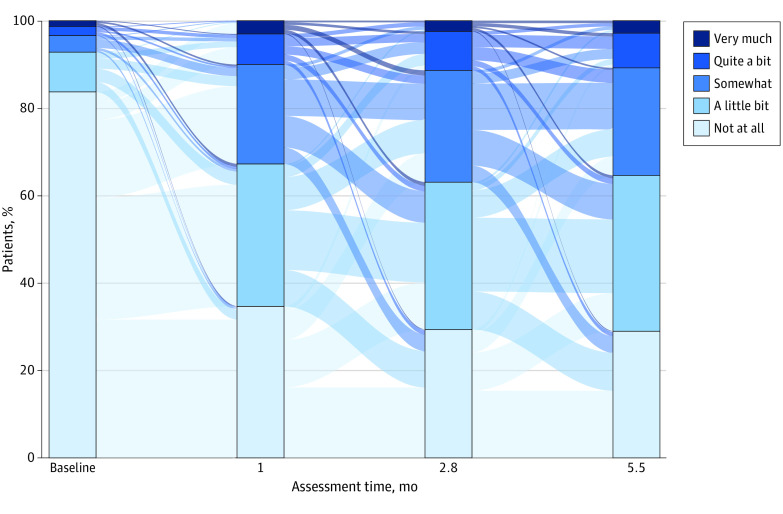
Sankey Bar Chart of GP5 Values The GP5 refers to item 5 of the Functional Assessment of Cancer Therapy-General. The sample sizes at each assessment time point included 988 patients at baseline, 953 patients at month, 895 patients at 2.8 months, and 631 patients at 5.5 months.

Figure 1 shows distributions of GP5 response options at each assessment time point. The vast majority reported “not at all” for baseline bother, but this proportion decreased substantially at each GP5 assessment while undergoing treatment. The time point with the largest proportion of participants responding either “somewhat,” “quite a bit,” or “very much” was at 2.8 months. The Sankey box plot in Figure 2 shows the change in GP5 values over each time point. Thick bands (indicating larger proportions) show large numbers of patients who reported “not at all” at baseline worsening to “a little bit” and “somewhat” at 1 month. Although the proportion of patients in each GP5 response category was largely stable from 1 month through 5.5 months, the Sankey plot demonstrates a subgroup of patients that were worsening as well as a separate subgroup of patients that improved at each time point. For example, a nontrivial amount of the patients reporting “a little bit” at 1 month worsened to “somewhat” (65 participants [24.3%]) at 2.8 months, while 61 (22.9%) improved to “not at all” at 2.8 months.

Table 2 shows the frequencies in each category for the GP5 while undergoing treatment and change indicators. The most frequent maximum value while undergoing treatment (ie, most severe) was “somewhat” for AE bother (352 participants [34.4%]). Using the baseline adjustment method, the most frequent maximum value while undergoing treatment was also “somewhat,” although it occurred less often than for the raw calculation (291 participants [30.6%]). A total of 264 patients (29.6%) worsened by 1 category from baseline to 1 month, 261 patients (31.1%) from baseline to 2 months, 197 patients (33.7%) from baseline to 5.5 months, and 276 patients from baseline to maximum GP5 value while undergoing treatment (29.0%); these proportions were similar for those who worsened by 2 or more categories. However, the proportion of patients who reported worsening by 2 or more categories when considering change from baseline to maximum GP5 while undergoing treatment was higher (464 participants [48.7%]).

**Table 2.** GP5 While Undergoing-Treatment and Change Indicators

Indicator	Participants, No./Total No. (%)
Maximum GP5 while undergoing treatment value	
Not at all	133/1022 (13.0)
A little bit	284/1022 (27.8)
Somewhat	352/1022 (34.4)
Quite a bit	168/1022 (16.4)
Very much	85/1022 (8.3)
Baseline-adjusted maximum GP5 value while undergoing treatment	
Not at all	212/952 (22.3)
A little bit	226/952 (23.7)
Somewhat	291/952 (30.6)
Quite a bit	149/952 (15.7)
Very much	74/952 (7.8)
Change from baseline to 1 mo	
Stayed the same or improved	394/891 (44.2)
Worsened by 1 category	264/891 (29.6)
Worsened by ≥2 categories	233/891 (26.2)
Change from baseline to 2.8 mos	
Stayed the same or improved	309/838 (36.9)
Worsened by 1 category	261/838 (31.1)
Worsened by ≥2 categories	268/838 (32.0)
Change from baseline to 5.5 mos	
Stayed the same or improved	219/584 (37.5)
Worsened by 1 category	197/584 (33.7)
Worsened by ≥2 categories	168/584 (28.8)
Change from baseline to maximum GP5 value while undergoing treatment	
Stayed the same or improved	212/952 (22.3)
Worsened by 1 category	276/952 (29.0)
Worsened by ≥2 categories	464/952 (48.7)

Abbreviation: GP5, Item 5 of the Functional Assessment of Cancer Therapy-General.

Table 3 shows unadjusted and adjusted ORs for logistic regressions of early treatment discontinuation due to AE for GP5 values while undergoing treatment (high vs low bother) and GP5 change from baseline. At 1 month, the odds of early discontinuation were more than twice as high for patients reporting high bother on the GP5 in comparison with those reporting low bother, even when adjusting for treatment group, age, baseline ECOG performance status, gender, race, and ISS stage (adjusted OR [aOR], 2.20; 95% CI, 1.25-3.89). The odds of early discontinuation for those reporting high bother on GP5 increased at 2.8 months (aOR, 3.41; 95% CI, 2.01-5.80) and at 5.5 months (aOR, 4.66; 95% CI, 1.69-12.83). The raw, maximum GP5 value while undergoing treatment was not associated with early treatment discontinuation, but the baseline-adjusted, maximum GP5 value while undergoing treatment was significantly associated with early treatment discontinuation for those with high bother compared with those with low bother (aOR, 1.54; 95% CI, 1.04-2.30). Regarding GP5 change from baseline, worsening of 2 or more categories was associated with higher odds of early discontinuation at 2.8 months (aOR, 3.02; 95% CI, 1.64-5.54) and 5.5 months (aOR, 5.49; 95% CI, 1.45-20.76). Worsening of 1 category was not associated with early discontinuation. Results were similar with and without adjustment for patient factors.

**Table 3.** Logistic Regression Models of Early Treatment Discontinuation and GP5 While UndergoingTreatment or Change From Baseline in GP5^a^

Indicator	Odds ratio (95% CI)	
	Unadjusted	Adjusted^b^
GP5 while undergoing treatment		
1-mo GP5 (high vs low bother)^c^	2.01 (1.17-3.45)	2.20 (1.25-3.89)
2.8-mo GP5 (high vs low bother)^c^	3.93 (2.38-6.49)	3.41 (2.01-5.80)
5.5-mo GP5 (high vs low bother)^c^	4.55 (1.75-11.84)	4.66 (1.69-12.83)
Maximum GP5 while undergoing treatment (high vs low bother)^c^	1.39 (0.94-2.05)	1.32 (0.89-1.98)
Baseline-adjusted, maximum GP5 while undergoing treatment (high vs low bother)^c^	1.61 (1.09-2.37)	1.54 (1.04-2.30)
Change from baseline to 1 month		
Worsened by 1 category vs stayed the same or improved	0.88 (0.54-1.43)	0.94 (0.57-1.54)
Worsened by ≥2 categories vs stayed the same or improved	1.38 (0.88-2.16)	1.41 (0.89-2.27)
Change from baseline to 2.8 months		
Worsened by 1 category vs stayed the same or improved	1.68 (0.88-3.20)	1.85 (0.95-3.59)
Worsened by ≥2 categories vs stayed the same or improved	3.17 (1.76-5.71)	3.02 (1.64-5.54)
Change from baseline to 5.5 months		
Worsened by 1 category vs stayed the same or improved	1.81 (0.43-7.69)	2.24 (0.51-9.88)
Worsened by ≥2 categories vs stayed the same or improved	5.36 (1.49-19.31)	5.49 (1.45-20.76)
Maximum change from baseline		
Worsened by 1 category vs stayed the same or improved	0.76 (0.43-1.35)	0.81 (0.45-1.48)
Worsened by ≥2 categories vs stayed the same or improved	1.35 (0.83-2.20)	1.40 (0.84-2.31)

Abbreviations: GP5, Item 5 of the Functional Assessment of Cancer Therapy-General; KRd, carfilzomib, lenalidomide, and dexamethasone.

^a^ Each row represents a separate logistic regression model. All models exclude patients with a GP5 value of “very much” at baseline.

^b^ Adjusts for treatment group (reference, KRd), age (entered as continuous), baseline Eastern Cooperative Oncology Group performance status (reference, 0), gender (reference, woman), race (reference, White), International Staging System stage (reference, Stage I).

^c^ High bother refers to responses of “very much”or “quite a bit” on the GP5, and low bother refers to responses of “somewhat,” “a little bit,” and “not at all” on the GP5.

eTables 1 to 9 in Supplement 1 show the results for each patient factor in the multivariable logistic regression models. In addition to GP5 while undergoing treatment and GP5 change indicators, treatment group (patients receiving VRd had higher odds of early discontinuation compared with patients receiving KRd) and patient age (older patients had higher odds of early discontinuation than younger patients) were significantly associated with early discontinuation due to AE in most, but not all, models. Treatment group was not associated with early discontinuation in models including the 5.5-month GP5 value and change from baseline to 5.5 months.

eTable 10 in Supplement 1 shows the results of the sensitivity analyses that added baseline GP5 as a covariate. The estimates resulting from this approach were very similar to those from our primary analysis that excluded patients with a GP5 response of “very much” at baseline.

## Discussion

In this survey study of the ECOG-ACRIN ENDURANCE trial,^12^ we found that the GP5 item performed well as an indicator of worsening tolerability of cancer treatment. Both changes from baseline in GP5, as well the GP5 values during treatment, were significantly associated with early treatment discontinuation due to AEs. These results point to the usefulness of GP5 as an overall AE impact measure for patients receiving treatment. Because it is very brief, GP5 may be very useful as a low-burden way to track treatment tolerability over time, both in the context of clinical trials and in routine care.

We operationalized GP5 in multiple ways to reflect the patient’s experience while receiving treatment, including mirroring PRO-CTCAE–based analyses (eg, the maximum GP5 value while undergoing treatment and change from baseline to the maximum value while undergoing treatment), as well as various magnitudes of change from baseline to GP5 while undergoing treatment assessments, and varying severity levels in GP5 while undergoing treatment. The most consistent associations with early treatment discontinuation were observed for high bother (ie, more severe bother) on the GP5 during treatment. Examining change from baseline, only worsening at a magnitude of 2 or more categories on GP5 was significantly associated with early treatment discontinuation. Together, these results indicate that a patient who reaches high-severity AE impact or who worsens in overall AE impact on GP5 is not tolerating their treatment well. In general, a patient’s maximum GP5 value while undergoing treatment, or change from baseline to this maximum value, was not a good indicator of early treatment discontinuation. This finding has implications for whether traditional methods used with CTCAE are appropriate for patient-reported tolerability data. Previous work^2,16,17^ has pointed out that the severity of patient-reported AE impact may not track linearly with increasing CTCAE grades.

In a trial comparing 2 aromatase inhibitors (ECOG-ACRIN E1Z03),^11^ researchers found that GP5 measured at baseline among postmenopausal women with receptor-positive primary breast cancer was associated with subsequent early treatment discontinuation. Most patients in the E1Z03 trial^11^ had received prior treatment, including prior adjuvant chemotherapy, raloxifene therapy, adjuvant radiotherapy, and hormonal therapy. Therefore, while that study^11^ showed that AEs carried over from previous treatment may impact a patient’s ability to tolerate a new treatment, it did not shed light on the ability of GP5 to capture the patient’s tolerability of a current treatment. The current study’s focus on GP5 responses from patients receiving treatment suggests that GP5 is potentially useful as a measure of tolerability while patients are receiving treatment in clinical trials. For example, in the current study, randomization to VRd was independently associated with increased odds of early treatment discontinuation due to AEs in most multivariable models, which may reflect the higher rate of peripheral neuropathy among VRd patients in comparison with the KRd group.^18^ Similar comparisons should be tested by positioning GP5 as a comparative tolerability end point in other trials.

The FDA and consensus reports^2,5^ have outlined a role for patient-reported tolerability data, including overall AE impact measures like GP5, as a complement to more traditional measures like CTCAE, dose reductions, and hospitalizations to form an overall picture of treatment tolerability. We note that overall AE impact complements symptomatic AEs measured by PROs like the PRO-CTCAE^19^ and single items from measurement systems like the Functional Assessment of Chronic Illness Therapy Item Library^6^ and the European Organization for Research and Treatment of Cancer Item Library.^20^ Therefore, we recommend administering GP5 along with PRO items on specific symptomatic AEs. Recent guidance suggests that overall AE impact and symptomatic AE PROs should be administered with high frequency through the early periods of a trial to capture rapidly emerging tolerability issues, perhaps as frequently as weekly.^5^ GP5 is feasible for high-frequency administration because it is only 1 item. Similarly, as new approaches and guidance emerge for use of PROs to capture tolerability in earlier-phase, dose-finding trials, GP5 is a promising candidate to efficiently capture impacts of toxic effects that are intolerable to patients.^21^ More empirical research is needed in this area.

### Limitations

Like any study, this report has limitations to consider. First, we used only 1 trial to examine the association of the AE experience of patients’ receiving treatment captured by GP5 with early treatment discontinuation. As such, our findings need to be tested and confirmed in other settings, including with other cancer types, other anticancer agents, and with a more sociodemographically diverse set of patient participants. In addition, it will be useful to examine whether similar results are found in a nontrial setting to determine whether GP5 will be useful for incorporation in clinical patient monitoring.^22,23^ Second, we note a concern about interpreting GP5 at a pretreatment assessment when a patient is naive to any systemic cancer treatment. A recent qualitative study^24^ found that, compared with patients receiving treatment, fewer patients who were treatment naive found GP5 to be relevant or understandable, and fewer reported being confident in their answers. In the current study, some patients were treatment naive at baseline. Therefore, we have less certainty about the meaning of GP5 responses of “a little bit” (86 participants), “somewhat” (58 participants), “quite a bit” (22 participants), and “very much” (13 participants) at baseline than we have for the same response at GP5 while undergoing treatment assessments. Third, we note that some patients were missing GP5 responses at each time point, even when they completed other items from the FACT-NTx or FACT-MM. While rates of missingness were low, ranging from 0% to 5% across the assessment time points, if missingness was nonrandom, there may be bias in our estimates of associations of GP5 while undergoing treatment with change in GP5 and early treatment discontinuation due to AE. While a previous study^25^ showed that postbaseline GP5 was not more likely to be missing than adjacent items as presented on the PRO form, future studies should investigate the mechanism of missingness for GP5 among patients in cancer trials.

## Conclusions

In conclusion, in this survey study, we report the GP5 item’s ability to capture treatment tolerability among a sample of patients with multiple myeloma. Our results indicate that patients who report high AE bother on the GP5 had higher odds of discontinuing treatment early. This study suggests that GP5 may be a useful, succinct way to track whether cancer patients tolerate their treatment and may identify patients vulnerable to tolerability-associated early discontinuation.

## Notes

Supplement 1. **eTable 1.** Multivariable Logistic Regression Analysis of Early Treatment Discontinuation on Month 1 GP5 and Patient Characteristics (N=872) **eTable 2.** Multivariable Logistic Regression Analysis of Early Treatment Discontinuation on GP5 Change From Baseline to Month 1 and Patient Characteristics (N=872) **eTable 3.** Multivariable Logistic Regression Analysis of Early Treatment Discontinuation on Month 2.8 GP5 and Patient Characteristics (N=823) **eTable 4**. Multivariable Logistic Regression Analysis of Early Treatment Discontinuation on GP5 Change From Baseline to Month 2.8 and Patient Characteristics (N=823) **eTable 5.** Multivariable Logistic Regression Analysis of Early Treatment Discontinuation on Month 5.5 GP5 and Patient Characteristics (N=574) **eTable 6.** Multivariable Logistic Regression Analysis of Early Treatment Discontinuation on GP5 Change From Baseline to Month 5.5 and Patient Characteristics (N=574) **eTable 7.** Multivariable Logistic Regression Analysis of Early Treatment Discontinuation on Maximum On-Treatment GP5 and Patient Characteristics (N=933) **eTable 8.** Multivariable Logistic Regression Analysis of Early Treatment Discontinuation on Baseline-Adjusted Maximum On-Treatment GP5 and Patient Characteristics (N=933) **eTable 9.** Multivariable Logistic Regression Analysis of Early Treatment Discontinuation on Maximum GP5 Change From Baseline and Patient Characteristics (N=933) **eTable 10.** Logistic Regression Models of Early Treatment Discontinuation on On-Treatment GP5 or Change From Baseline in GP5 with Baseline GP5 Adjustment

Supplement 2.
Data Sharing Statement
