# Supplementary material for: Patient-Reported Adverse Events and Early Treatment Discontinuation Among Patients With Multiple Myeloma
Source: JAMA Netw Open. 2024 Mar 27;7(3):e243854. doi: 10.1001/jamanetworkopen.2024.3854 (PMC10973895; doi:10.1001/jamanetworkopen.2024.3854)
Supplement: Supplement 1. — eTable 1. Multivariable Logistic Regression Analysis of Early Treatment Discontinuation on Month 1 GP5 and Patient Characteristics (N=872) eTable 2. Multivariable Logistic Regression Analysis of Early Treatment Discontinuation on GP5 Change From Baseline to Month 1 and Patient Characteristics (N=872) eTable 3. Multivariable Logistic Regression Analysis of Early Treatment Discontinuation on Month 2.8 GP5 and Patient Characteristics (N=823) eTable 4. Multivariable Logistic Regression Analysis of Early Treatment Discontinuation on GP5 Change From Baseline to Month 2.8 and Patient Characteristics (N=823) eTable 5. Multivariable Logistic Regression Analysis of Early Treatment Discontinuation on Month 5.5 GP5 and Patient Characteristics (N=574) eTable 6. Multivariable Logistic Regression Analysis of Early Treatment Discontinuation on GP5 Change From Baseline to Month 5.5 and Patient Characteristics (N=574) eTable 7. Multivariable Logistic Regression Analysis of Early Treatment Discontinuation on Maximum On-Treatment GP5 and Patient Characteristics (N=933) eTable 8. Multivariable Logistic Regression Analysis of Early Treatment Discontinuation on Baseline-Adjusted Maximum On-Treatment GP5 and Patient Characteristics (N=933) eTable 9. Multivariable Logistic Regression Analysis of Early Treatment Discontinuation on Maximum GP5 Change From Baseline and Patient Characteristics (N=933) eTable 10. Logistic Regression Models of Early Treatment Discontinuation on On-Treatment GP5 or Change From Baseline in GP5 with Baseline GP5 Adjustment [file jamanetwopen-e243854-s001.pdf]

Supplemental Online Content

Peipert JD, Zhao F, Lee J, et al. Patient-reported adverse events and early treatment discontinuation among patients with multiple myeloma: a secondary analysis of the ECOG-ACRIN E1A11 trial. *JAMA Netw Open.* 2024;7(3):e243854. doi:10.1001/jamanetworkopen.2024.3854

eTable 1. Multivariable Logistic Regression Analysis of Early Treatment Discontinuation on Month 1 GP5 and Patient Characteristics (N=872)

eTable 2. Multivariable Logistic Regression Analysis of Early Treatment Discontinuation on GP5 Change From Baseline to Month 1 and Patient Characteristics (N=872)

eTable 3. Multivariable Logistic Regression Analysis of Early Treatment Discontinuation on Month 2.8 GP5 and Patient Characteristics (N=823)

eTable 4 . Multivariable Logistic Regression Analysis of Early Treatment Discontinuation on GP5 Change From Baseline to Month 2.8 and Patient Characteristics (N=823)

eTable 5. Multivariable Logistic Regression Analysis of Early Treatment Discontinuation on Month 5.5 GP5 and Patient Characteristics (N=574)

eTable 6. Multivariable Logistic Regression Analysis of Early Treatment Discontinuation on GP5 Change From Baseline to Month 5.5 and Patient Characteristics (N=574)

eTable 7. Multivariable Logistic Regression Analysis of Early Treatment Discontinuation on Maximum On-Treatment GP5 and Patient Characteristics (N=933)

eTable 8. Multivariable Logistic Regression Analysis of Early Treatment Discontinuation on Baseline-Adjusted Maximum On-Treatment GP5 and Patient Characteristics (N=933)

eTable 9. Multivariable Logistic Regression Analysis of Early Treatment Discontinuation on Maximum GP5 Change From Baseline and Patient Characteristics (N=933)

eTable 10. Logistic Regression Models of Early Treatment Discontinuation on On-Treatment GP5 or Change From Baseline in GP5 with Baseline GP5 Adjustment

© 2024 Piepert JD et al. *JAMA Network Open.*

**eTable 1. Multivariable Logistic Regression Analysis of Early Treatment Discontinuation on Month 1 GP5 and Patient Characteristics (N=872)**	
	**Odds Ratio (95% CI)**
High Bother on Month 1 GP5[a]	2.20 (1.25, 3.89)
Treatment Arm (VRd vs. KRd)	1.71 (1.14, 2.57)
Age (entered as continuous)	1.07 (1.04, 1.10)
Baseline ECOG performance status	
1 vs. 0	1.51 (0.98, 2.33)
2 vs. 0	1.60 (0.71, 3.58)
3 vs. 0	1.68 (0.34, 8.23)
Gender: Male vs. Female	0.85 (0.57, 1.27)
Race	
Black/African American vs. White	1.59 (0.90, 2.83)
Asian vs. White	0.62 (0.08, 5.03)
Other Race/Not Reported vs. White	0.82 (0.28, 2.42)
International Staging System	
II vs. I	0.85 (0.53, 1.36)
III vs. I	0.71 (0.42, 1.22)
Model c-statistic	0.69
Analytical Note: Excludes patients with GP5 values of Very Much or Quite a bit at baseline.<br>[a]High bother = Very much, Quite a bit; Low bother = Somewhat, A Little Bit, Not at All	

© 2024 Piepert JD et al. *JAMA Network Open.*

**eTable 2. Multivariable Logistic Regression Analysis of Early Treatment Discontinuation on GP5 Change From Baseline to Month 1 and Patient Characteristics (N=872)**	
	**Odds Ratio (95% CI)**
GP5 Change from Baseline to Month 1	
1 category worsening vs. stayed the same/improved	0.94 (0.57, 1.54)
≥2 categories worsening vs. stayed the same/improved	1.41 (0.89, 2.27)
Treatment Arm (VRd vs. KRd)	1.71 (1.14, 2.57)
Age (entered as continuous)	1.07 (1.04, 1.09)
Baseline ECOG performance status	
1 vs. 0	1.52 (0.98, 2.34)
2 vs. 0	1.54 (0.69, 3.45)
3 vs. 0	1.45 (0.29, 7.17)
Gender: Male vs. Female	0.87 (0.58, 1.30)
Race	
Black/African American vs. White	1.59 (0.90, 2.83)
Asian vs. White	0.57 (0.07, 4.65)
Other Race/Not Reported vs. White	0.85 (0.29, 2.48)
International Staging System	
II vs. I	0.87 (0.55, 1.39)
III vs. I	0.74 (0.43, 1.26)
Model c-statistic	0.69
Analytical Note: Excludes patients with GP5 values of Very Much or Quite a bit at baseline. [a]High bother = Very much, Quite a bit; Low bother = Somewhat, A Little Bit, Not at All	

© 2024 Piepert JD et al. *JAMA Network Open.*

**eTable 3. Multivariable Logistic Regression Analysis of Early Treatment Discontinuation on Month 2.8 GP5 and Patient Characteristics (N=823)**	
	**Odds Ratio (95% CI)**
High Bother on Month 2.8 GP5[a]	3.41 (2.01, 5.80)
Treatment Arm (VRd vs. KRd)	2.27 (1.38, 3.74)
Age (entered as continuous)	1.06 (1.03, 1.09)
Baseline ECOG performance status	
1 vs. 0	1.59 (0.95, 2.66)
2 vs. 0	1.56 (0.59, 4.08)
3 vs. 0	NE
Gender: Male vs. Female	0.92 (0.57, 1.49)
Race	
Black/African American vs. White	1.43 (0.72, 2.84)
Asian vs. White	NE
Other Race/Not Reported vs. White	0.80 (0.23, 2.82)
International Staging System	
II vs. I	0.83 (0.48, 1.44)
III vs. I	0.70 (0.37, 1.34)
Model c-statistic	0.73

Analytical Note: Excludes patients with GP5 values of Very Much or Quite a bit at baseline.
[a]High bother = Very much, Quite a bit; Low bother = Somewhat, A Little Bit, Not at All
NE: Not estimable due to low number of events in one of the covariate categories.

© 2024 Piepert JD et al. *JAMA Network Open.*

eTable 4. Multivariable Logistic Regression Analysis of Early Treatment Discontinuation on GP5 Change From Baseline to Month 2.8 and Patient Characteristics (N=823)

	**Odds Ratio (95% CI)**
GP5 Change from Baseline to Month 2.8	
1 category worsening vs. stayed the same/improved	1.85 (0.95, 3.59)
≥2 categories worsening vs. stayed the same/improved	3.02 (1.64, 5.54)
Treatment Arm (VRd vs. KRd)	2.38 (1.45, 3.90)
Age (entered as continuous)	1.06 (1.03, 1.10)
Baseline ECOG performance status	
1 vs. 0	1.69 (1.01, 2.83)
2 vs. 0	1.38 (0.53, 1.50)
3 vs. 0	NE
Gender: Male vs. Female	0.93 (0.58, 1.50)
Race	
Black/African American vs. White	1.54 (0.79, 3.03)
Asian vs. White	NE
Other Race/Not Reported vs. White	0.89 (0.25, 3.14)
International Staging System	
II vs. I	0.87 (0.50, 1.50)
III vs. I	0.66 (0.35, 1.26)
Model c-statistic	0.73

Analytical Note: Excludes patients with GP5 values of Very Much or Quite a bit at baseline.
[a]High bother = Very much, Quite a bit; Low bother = Somewhat, A Little Bit, Not at All
NE: Not estimable due to low number of events in one of the covariate categories.

© 2024 Piepert JD et al. *JAMA Network Open.*

eTable 5. Multivariable Logistic Regression Analysis of Early Treatment Discontinuation on Month 5.5 GP5 and Patient Characteristics (N=574)	
	**Odds Ratio (95% CI)**
High Bother on Month 5.5 GP5[a]	4.66 (1.69, 12.83)
Treatment Arm (VRd vs. KRd)	1.68 (0.66, 4.27)
Age (entered as continuous)	1.08 (1.02, 1.15)
Baseline ECOG performance status	
1 vs. 0	1.53 (0.57, 4.13)
2 vs. 0	2.50 (0.46, 13.66)
3 vs. 0	NE
Gender: Male vs. Female	1.18 (0.44, 3.16)
Race	
Black/African American vs. White	0.90 (0.19, 4.23)
Asian vs. White	NE
Other Race/Not Reported vs. White	1.29 (0.15, 10.98)
International Staging System	
II vs. I	0.23 (0.07, 0.79
III vs. I	0.24 (0.06, 0.92)
Model c-statistic	0.81

Analytical Note: Excludes patients with GP5 values of Very Much or Quite a bit at baseline.
[a]High bother = Very much, Quite a bit; Low bother = Somewhat, A Little Bit, Not at All
NE: Not estimable due to low number of events in one of the covariate categories.

© 2024 Piepert JD et al. *JAMA Network Open.*

**eTable 6. Multivariable Logistic Regression Analysis of Early Treatment Discontinuation on GP5 Change From Baseline to Month 5.5 and Patient Characteristics (N=574)**	
	**Odds Ratio (95% CI)**
GP5 Change from Baseline to Month 5.5	
1 category worsening vs. stayed the same/improved	2.24 (0.51, 9.88)
>2 categories worsening vs. stayed the same/improved	5.49 (1.45, 20.76)
Treatment Arm (VRd vs. KRd)	1.48 (0.58, 3.80)
Age (entered as continuous)	1.08 (1.02, 1.15)
Baseline ECOG performance status	
1 vs. 0	1.65 (0.60, 4.48)
2 vs. 0	3.15 (0.57, 17.31)
3 vs. 0	NE
Gender: Male vs. Female	1.25 (0.47, 3.33)
Race	
Black/African American vs. White	0.97 (0.20, 4.66)
Asian vs. White	NE
Other Race/Not Reported vs. White	1.30 (0.15, 11.30)
International Staging System	
II vs. I	0.23 (0.07, 0.77)
III vs. I	0.24 (0.06, 0.92)
Model c-statistic	0.80
Analytical Note: Excludes patients with GP5 values of Very Much or Quite a bit at baseline.<br>[a]High bother = Very much, Quite a bit; Low bother = Somewhat, A Little Bit, Not at All<br>NE: Not estimable due to low number of events in one of the covariate categories.	

© 2024 Piepert JD et al. *JAMA Network Open.*

**eTable 7. Multivariable Logistic Regression Analysis of Early Treatment Discontinuation on Maximum On-Treatment GP5 and Patient Characteristics (N=933)**	
	**Odds Ratio (95% CI)**
High Bother on Maximum On-Treatment GP5[a]	1.32 (0.89, 1.98)
Treatment Arm (VRd vs. KRd)	1.83 (1.23, 2.73)
Age (entered as continuous)	1.06 (1.04, 1.09)
Baseline ECOG performance status	
1 vs. 0	1.53 (1.01, 2.34)
2 vs. 0	1.41 (0.63, 3.13)
3 vs. 0	1.27 (0.27, 6.08)
Gender: Male vs. Female	0.91 (0.61, 1.34)
Race	
Black/African American vs. White	1.38 (0.78, 2.43)
Asian vs. White	0.52 (0.06, 4.21)
Other Race/Not Reported vs. White	0.82 (0.28, 2.39)
International Staging System	
II vs. I	0.91 (0.58, 1.44)
III vs. I	0.76 (0.45, 1.28)
Model c-statistic	0.68
Analytical Note: Excludes patients with GP5 values of Very Much or Quite a bit at baseline.<br>[a]High bother = Very much, Quite a bit; Low bother = Somewhat, A Little Bit, Not at All	

© 2024 Piepert JD et al. *JAMA Network Open.*

**eTable 8. Multivariable Logistic Regression Analysis of Early Treatment Discontinuation on Baseline-Adjusted Maximum On-Treatment GP5 and Patient Characteristics (N=933)**	
	**Odds Ratio (95% CI)**
High Bother on Baseline-Adjusted Maximum On-Treatment GP5[a]	1.54 (1.04, 2.30)
Treatment Arm (VRd vs. KRd)	1.83 (1.23, 2.73)
Age (entered as continuous)	1.06 (1.04, 1.09)
Baseline ECOG performance status	
1 vs. 0	1.54 (1.01, 2.35)
2 vs. 0	1.43 (0.65, 3.19)
3 vs. 0	1.32 (0.27, 6.31)
Gender: Male vs. Female	0.90 (0.61, 1.33)
Race	
Black/African American vs. White	1.37 (0.77, 2.41)
Asian vs. White	0.54 (0.07, 4.36)
Other Race/Not Reported vs. White	0.81 (0.28, 2.37)
International Staging System	
II vs. I	0.92 (0.58, 1.44)
III vs. I	0.76 (0.45, 1.28)
Model c-statistic	0.68
Analytical Note: Excludes patients with GP5 values of Very Much or Quite a bit at baseline.<br>[a]High bother = Very much, Quite a bit; Low bother = Somewhat, A Little Bit, Not at All	

© 2024 Piepert JD et al. *JAMA Network Open.*

**eTable 9. Multivariable Logistic Regression Analysis of Early Treatment Discontinuation on Maximum GP5 Change From Baseline and Patient Characteristics (N=933)**	
	**Odds Ratio (95% CI)**
Maximum GP5 Change from Baseline	
1 category worsening vs. stayed the same/improved	0.81 (0.45, 1.48)
≥2 categories worsening vs. stayed the same/improved	1.40 (0.84, 2.31)
Treatment Arm (VRd vs. KRd)	1.85 (1.24, 2.76)
Age (entered as continuous)	1.06 (1.04, 1.09)
Baseline ECOG performance status	
1 vs. 0	1.54 (1.01, 2.36)
2 vs. 0	1.43 (0.65, 3.19)
3 vs. 0	1.25 (0.26, 6.02)
Gender: Male vs. Female	0.90 (0.61, 1.34)
Race	
Black/African American vs. White	1.36 (0.77, 2.40)
Asian vs. White	0.52 (0.06, 4.24)
Other Race/Not Reported vs. White	0.80 (0.27, 2.34)
International Staging System	
II vs. I	0.91 (0.58, 1.44)
III vs. I	0.78 (0.46, 1.31)
Model c-statistic	0.69
Analytical Note: Excludes patients with GP5 values of Very Much or Quite a bit at baseline. [a]High bother = Very much, Quite a bit; Low bother = Somewhat, A Little Bit, Not at All	

© 2024 Piepert JD et al. *JAMA Network Open.*

**eTable 10. Logistic Regression Models of Early Treatment Discontinuation on On-Treatment GP5 or Change From Baseline in GP5 with Baseline GP5 Adjustment**		
Note: Each row represents a separate logistic regression model.	**Baseline GP5 Adjusted Odds Ratio (95% CI)**	**Fully Adjusted Odds Ratio[b] (95% CI)**
**On-Treatment GP5**		
Month 1 GP5 (High vs Low Bother) [a]	**1.96 (1.15, 3.35)**	**2.13 (1.21, 3.75)**
Month 2.8 GP5 (High vs Low Bother) [a]	**3.86 (2.36, 6.33)**	**3.45 (2.05, 5.81)**
Month 5.5 GP5 (High vs Low Bother) [a]	**4.84 (1.95, 12.05)**	**4.97 (1.89, 13.03)**
Maximum On-Treatment GP5 (High vs Low Bother) [a]	1.44 (0.97, 2.12)	1.36 (0.91, 2.03)
Baseline-Adjusted Maximum On-Treatment GP5 (High vs Low Bother) [a]	**N/A**	**N/A**
**Change from Baseline in GP5**		
Change from Baseline to Month 1		
1 category worsening vs. stayed the same/improved	0.87 (0.54, 1.42)	0.93 (0.56, 1.54)
≥2 categories worsening vs. stayed the same/improved	1.38 (0.86, 2.19)	1.43 (0.88, 2.33)
Change from Baseline to Month 2.8		
1 category worsening vs. stayed the same/improved	1.72 (0.89, 3.35)	1.92 (0.97, 3.81)
≥2 categories worsening vs. stayed the same/improved	**3.34 (1.78, 6.25)**	**3.24 (1.70, 6.16)**
Change from Baseline to Month 5.5		
1 category worsening vs. stayed the same/improved	1.61 (0.40, 6.44)	1.98 (0.47, 8.30)
≥2 categories worsening vs. stayed the same/improved	**5.02 (1.41, 17.93)**	**5.45 (1.44, 20.50)**
Maximum Change from Baseline		
1 category worsening vs. stayed the same/improved	0.75 (0.41, 1.37)	0.81 (0.44, 1.49)
≥2 categories worsening vs. stayed the same/improved	1.37 (0.80, 2.34)	1.43 (0.83, 2.46)

Analytical Note: Change from baseline in GP5 analyses excludes patients with a GP5 value of Very Much at baseline.

[a] High bother = Very much, Quite a bit; Low bother = Somewhat, A Little Bit, Not at All

[b] Adjusts for baseline GP5, treatment arm (reference = KRd), age (entered as continuous), baseline ECOG performance status (reference = 0), gender (reference = female), race (reference = White), international staging system stage (reference = Stage I)

© 2024 Piepert JD et al. *JAMA Network Open.*
