# Supplementary material for: Patient-Reported Adverse Events and Early Treatment Discontinuation Among Patients With Multiple Myeloma
Source: JAMA Netw Open. 2024 Mar 27;7(3):e243854. doi: 10.1001/jamanetworkopen.2024.3854 (PMC10973895; doi:10.1001/jamanetworkopen.2024.3854)
Supplement: Supplement 2. — Data Sharing Statement [file jamanetwopen-e243854-s002.pdf]

Data Sharing Statement

Peipert. Patient-Reported Adverse Events and Early Treatment Discontinuation Among Patients With Multiple Myeloma: A Secondary Analysis of the ECOG-ACRIN E1A11 Trial.
JAMA Netw Open. Published March 27, 2024. doi:10.1001/jamanetworkopen.2024.3854

Data
Data available: Yes
Data types: Deidentified participant data
How to access data: Requests for access to the dataset can be made to the NCTN/NCORP data archive (https://nctn-data-archive.nci.nih.gov).
When available: beginning date: 08-25-2023

Supporting Documents
Document types: None

Additional Information
Who can access the data: Individuals with an account on the NCTN/NCORP data archive can make a request.
Types of analyses: Specified purpose.
Mechanisms of data availability: With approval of the NCTN/NCORP data archive.
